# Efficacy of mepolizumab treatment in oral corticosteroid-dependent severe eosinophilic asthma patients with chronic rhinosinusitis with nasal polyps: single center, real life study

**DOI:** 10.3906/sag-1912-62

**Published:** 2020-04-09

**Authors:** İnsu YILMAZ, Murat TÜRK, Sakine BAHÇECİOĞLU, Nuri TUTAR, İnci GÜLMEZ

**Affiliations:** 1 Division of Immunology and Allergy, Department of Chest Diseases, Faculty of Medicine, Erciyes University, Kayseri Turkey; 2 Department of Chest Diseases, Faculty of Medicine, Erciyes University, Kayseri Turkey

**Keywords:** Severe asthma, eosinophilic asthma, chronic rhinosinusitis, nasal polyps, mepolizumab, anti-IL5

## Abstract

**Background/aim:**

Oral corticosteroid (OCS)-dependent severe eosinophilic asthma with chronic rhinosinusitis with nasal polyps (SEA-CRSwNP) would be a suitable phenotype for mepolizumab treatment. This study evaluated the short-term efficacy of mepolizumab treatment in OCS-dependent SEA-CRSwNP.

**Materials and methods:**

Baseline and 24th week results [daily OCS doses, asthma exacerbation frequency, asthma control test (ACT) scores, blood eosinophil levels, FEV1 values, and numerical analog scale (NAS) of CRSwNP symptoms] of patients who were treated for at least 24 weeks with mepolizumab were retrospectively evaluated and compared.

**Results:**

A total of 16 patients were enrolled in the study. Mepolizumab was discontinued in one patient due to side effects. The daily OCS dosage was reduced from baseline in all patients, and at week 24 OCS was discontinued in 40% of the patients (baseline mean steroid dose: 9.2 ± 5.2 mg, 24th week: 1.3 ± 1.4 mg; P < 0.001). The number of asthma exacerbations within 24 weeks significantly decreased after beginning mepolizumab treatment (2.1 ± 2.7 vs. 0.07 ± 0.26; P = 0.012), and a significant increase in ACT scores (baseline mean ACT: 18 ± 5.7; 24th week mean ACT: 23.3 ± 3; P = 0.006) was observed despite the decrease in daily OCS dosages. There was no significant difference in FEV1 values between baseline and week 24. Evaluation of the general symptoms of CRSwNP, as per NAS, revealed that the baseline mean NAS was 5.6 ± 4.4, and the 24th week mean NAS was 3.2 ± 3.2 (P = 0.021).

**Conclusion:**

This is the first real-life study evaluating the short-term efficacy of mepolizumab treatment on OCS-dependent SEA-CRSwNP. This study demonstrates that mepolizumab is an effective and safe biologic for the treatment of this severe asthma subphenotype.

## 1. Introduction

The anti-IL5 antibody mepolizumab was approved in 2015 and since then has become an established therapy for patients with severe uncontrolled eosinophilic asthma Difficult-to-treat and severe asthma in adolescent and adult patients. Diagnosis and management. The Global Initiative for Asthma 2019. [online]. Website: https://ginasthma.org/severeasthma/ (17 september 2019, date last accessed). In the earliest studies, mepolizumab was administered to a nonspecific population of patients with moderate, persistent asthma and variable levels of eosinophilia. Although no significant clinical results have been reported for asthma control from the earliest studies, its effectiveness in eosinophilic asthma is now well documented [1,2]. Clinical trials have shown that mepolizumab displays high clinical efficacy by reducing exacerbation rates and daily oral corticosteroid (OCS) intake and enhancing quality of life. It is apparent that careful selection of patients is required in order to achieve the best results [3–5]. 

Among the several different asthma phenotypes, eosinophilic inflammation occurs in more than 50% of patients with either atopic or nonatopic asthma. High eosinophil counts, in both peripheral blood and the airways, are associated with recurrent disease exacerbations and severe airflow limitation [6]. Adult-onset eosinophilic asthma is increasingly recognized as one of the most severe asthma phenotypes [7–10]. Another characteristic feature of adult-onset eosinophilic asthma is comorbid chronic rhinosinusitis with nasal polyps (CRSwNP), a feature known for many years and, in some cases, linked with aspirin and other nonsteroidal antiinflammatory drug hypersensitivities [11–13]. Concern regarding nonallergic, severe eosinophilic asthma and its associated comorbidities has increased with the awareness that this subtype is characterized by high levels of the proeosinophilic cytokine IL-5, which is mainly produced by a unique population of type 2 innate lymphoid cells [14]. We endorse an in-house classification of asthma phenotypes and a decision-making protocol which involves the first choice monoclonal antibodies (mAbs) and potential alternative mAbs in severe asthma phenotypes [11]. We prefer anti-IL5 treatment, especially in the OCS-dependent severe eosinophilic asthma with CRSwNP (SEA-CRSwNP) phenotype. 

To the best of our knowledge, no study evaluating the real-life efficacy of mepolizumab treatment in patients with OCS-dependent SEA-CRSwNP phenotype has been carried out to date. According to the literature, data regarding postmarketing studies that have evaluated the effects of mepolizumab in real-world settings are scarce [15,16]. Thus, this short-term single-center study was carried out in a more specific group of asthmatic patients treated with add-on biologic therapy with mepolizumab.

## 2. Methods

Adult patients (>18 years) with OCS-dependent SEA-CRSwNP phenotype who were treated with mepolizumab between 2018 and 2019 were retrospectively evaluated. All patients were treated with high-dose, extrafine inhaled glucocorticoids (ICS) and a long-acting β2-agonist, along with a second controller montelukast in addition to regular OCS therapy at least 6 months before mepolizumab treatment. Indications for treatment with mepolizumab were approved on the basis of the Turkish Social Security Institution Health Application Communique, according to which, mepolizumab can be administered to patients with severe eosinophilic asthma who have: a) blood eosinophil count ≥300 cells/µL (≥150 cells/µL if the patient is under long-term, regular OCS therapy) and b) controlled or uncontrolled asthma treated with regular systemic steroids for at least 6 months and/or uncontrolled asthma (approximately two attacks per year requiring systemic corticosteroids for at least 3 days) despite use of a high combination dosage of ICS (>800 µg/day budesonide, or equivalent) and long-acting inhaled β2 agonist for at least one year Turkish Social Security Institution Health Application Communique [online]. Website: http://www.mevzuat.gov.tr/Metin.Aspx?MevzuatKod=9.5.17229&MevzuatIliski=0&sourceXmlSearch= (25 september 2019, date last accessed). However, we have reduced the criterion, and mepolizumab was employed only in patients with GINA step 5 OCS-dependent SEA-CRSwNP (uncontrolled, partially controlled, or, in cases where OCS side effects developed, even in cases of controlled asthma). Mepolizumab was administered subcutaneously at a dose of 100 mg every four weeks for at least 12 weeks. Mepolizumab was continued if there was a clinical response at week 12. Those patients who completed at least 24 weeks of treatment were included in the study.

Throughout the study period parameters including ACT score, blood eosinophil count, and FEV1 were measured at baseline, week 12, and week 24 after the first injection of mepolizumab. In addition, the number of asthma exacerbations (exacerbations occurring within the previous 24 weeks) and daily intake of OCS (presented as methyl-prednisone equivalent in milligrams) were also recorded, respectively, at baseline and at week 24 of mepolizumab treatment. 

The scores for severity of nasal polyposis on the numerical analog scale (NAS) were assessed by asking patients to indicate the severity of their CRSwNP using a 0 to 10 scale (0: no symptoms; 10: worst symptoms possible) and the irritability of the following symptoms in general: rhinorrhea, mucus in the throat, nasal blockage, and loss of smell. The NAS score for the loss of smell was evaluated separately. Changes in the severity of nasal polyposis according to NAS score from baseline to the 24th week were also recorded. In addition, the side effects of mepolizumab were assessed for all patients.

### 2.1. Differentiation of severe asthma from difficult asthma

Drug adherence; inhaler technique; and comorbidities including allergic rhinitis, chronic rhinosinusitis/nasal polyps, gastroesophageal reflux, obstructive sleep apnea syndrome; and trigger factors like allergens, smoking, occupational allergens and/or irritants, ACE inhibitors, and nonspecific beta-blockers which interfered with disease control were investigated for all patients. Measures taken for these comorbidities and triggers, drug compliance, and techniques were analyzed. All patients under GINA step 5 treatment with controlled or uncontrolled asthma were considered to be severely asthmatic, and OCS-dependent SEA-CRSwNP was identified as the appropriate asthma phenotype for mepolizumab treatment in these patients.

Since this asthma phenotype is associated with high eosinophilia, other diseases associated with higher peripheral eosinophilia and accompanied by asthma or asthma-like symptoms, such as eosinophilic granulomatosis with polyangiitis, allergic bronchopulmonary aspergillosis, chronic eosinophilic pneumonia, hypereosinophilic syndrome, Loffler’s syndrome, and pulmonary involvement of connective tissue diseases were ruled out.

### 2.2. Definitions

### 2.2.1, Asthma exacerbations

An exacerbation was defined as a worsening of asthma symptoms requiring OCS at least three days a week or an increase in the OCS dose. 

#### 2.2.2. Chronic rhinosinusitis (CRS)

All CRS subjects met the criteria for CRS as defined by the American Academy of Otolaryngology–Head and Neck Surgery Chronic Rhinosinusitis Task Force. The diagnosis of CRS was based on the presence of clinical symptoms (i.e. nasal congestion, rhinorrhea, facial pressure, and hyposmia) for more than 12 weeks in addition to the objective evidence of chronic inflammatory disease on sinus CT imaging or nasal endoscopy. Sinonasal involvement was assessed by paranasal sinus computerized tomography (PNCT) and nasal endoscopy [17]. 

#### 2.2.3. CRSwNP

CRSwNP is characterized by the occurrence for more than 12 weeks of symptoms such as nasal discharge, stuffiness, facial pressure or pain, dysfunction or loss of the sense of smell, and cough from postnasal drip and by the polypoid inflammation filling the nasal airway in the PNCT [18]. 

#### 2.2.4. NAS

In NAS, the response to “How troublesome are your CRSwNP symptoms?” is rated from 0 to 10 (0 = not troublesome, 10 = worst symptoms possible).

#### 2.2.5. Treatment response to mepolizumab

Based on placebo-controlled, phase III studies, recommendations published by the National Institute for Health and Care Excellence (NICE) define the reduction of the exacerbation rate by at least 50%, or a clinically significant reduced dose of continuous OCS, as adequate response criteria [4,5,] Mepolizumab for treating severe refractory eosinophilic asthma. National Institute for Health and Care Excellence (NICE) [online]. Website: https://www.nice.org.uk/guidance/ta431 (19 July 2019, date last accessed). 

### 2.3. Laboratory, functional, and imaging tests

The tests included blood eosinophilia (reference range: <200 cells/mm3), C-reactive protein (CRP; reference range: 0–6 mg/L), erythrocyte sedimentation rate (ESR; reference range: 3–20 mm/h), total immunoglobulin E (IgE; reference range: 0–100 IU/mL), *Aspergillus* specific IgE, antinuclear antibody, urinalysis, liver and renal function tests, parasite stool examination, creatine kinase, pulmonary function tests [including FEV1, forced vital capacity (FVC), and FEV1/FVC], thorax computed tomography, PNCT, and electromyography if the patients exhibited symptoms of peripheral neuropathy. The authors also requested advanced laboratory tests for eosinophilic granulomatosis with polyangiitis (EGPA), hypereosinophilic syndrome (HES), and lymphoreticular malignancy among patients who had >10% blood eosinophils (such as vitamin B12, antineutrophil cytoplasmic antibody, troponin, FIP1-like-1platelet-derived growth factor receptor alpha, JAK-2 mutation, and Philadelphia chromosome; abdominal ultrasonography was carried out if suggested by hematologic consultation). 

### 2.4. Glucocorticoid reduction phase scheme

The dose of methylprednisone was reduced every 4 weeks according to a predefined schedule (Table 1) if the patient had not had an exacerbation with a decrease in ACT score. In patients who were receiving a daily dose of 8 mg or more of methylprednisone at baseline, the dose of the drug was not reduced to zero without consulting endocrinology due to concern regarding withdrawal effects.

**Table 1:** Glucocorticoid reduction phase scheme.

Methylprednisolone Dose (mg/day)						
20.0	16.0	12.0	10.0	8.0	6.0
4.0	16.0	12.0	10.0	8.0	6.0	4.0
2.0	12.0	10.0	8.0	6.0	4.0	2.0
2.0*	10.0	8.0	6.0	4.0	2.0	2.0*
0.0	8.0	6.0	4.0	2.0	2.0*	0.0
0.0	6.0	4.0	2.0	2.0*	0.0	0.0
0.0	4.0	2.0	2.0*	0.0	0.0	0.0
0.0	2.0	2.0*	0.0	0.0	0.0	0.0
0.0	2.0*	0.0	0.0	0.0	0.0	0.0
0.0

*Taken as 2.0 mg administered every other day.

All patients under follow-up at our asthma outpatient clinic provided written informed consent. Ethics approval was obtained from the Erciyes University ethics committee (approval date and number: 12 August; 2019-20019/472).

### 2.5. Statistical analysis

Data were entered into SPSS software version 17.0 (SPSS Inc., Chicago, IL, USA), and analyses were made using the same program. All continuous variables were presented as mean ± standard deviation (SD) due to the small sample size. For all nonparametric variables between and within groups, comparisons were made using the Mann–Whitney U-test and Wilcoxon test, respectively. P values <0.05 were considered significant in all analyses.

## 3. Results

Data from 16 patients with OCS-dependent SEA-CRSwNP who underwent treatment with mepolizumab were analyzed. All patients were classified as step 5 according to the Global Initiative for Asthma (GINA)1 and had uncontrolled asthma despite maximal therapy. The mean age of the patients was 48.6 ± 11.9 years. The mean duration of the disease and the duration of regular OCS use prior to the initiation of mepolizumab treatment were 12.9 ± 6.6 years and 5.1 ± 2.6 years, respectively. Females accounted for 81% of all the study subjects. Of the 16 patients, 14 (88%) were nonsmokers. Patient characteristics are shown in Table 2. 

**Table 2:** Characteristics of the patients.

	N = 16
Females (%)	13 (81)
Age, years, mean ± SD	48.6 ± 11.9
Smoking story (%)Never smokedEx-smokerActive smoker	14 (88)1 (6)1 (6)
Asthma duration, years, mean ± SD	12.9 ± 6.6
Mean clinical follow-up duration, years ± SD	5.1 ± 2.6
NERD (%)	10 (63)
Atopy (%)	3 (19)
Baseline total IgE levels, IU/mL, mean ± SD	545 ± 977

NERD: NSAID-exacerbated respiratory disease.

Disease control was evaluated using ACT at baseline with a mean value of 18.2 ± 5.5. In addition, a lung function test prior to mepolizumab treatment evidenced a mean FEV1 of 81% ± 30. All patients were receiving daily OCS therapy before mepolizumab treatment (a mean dose of 8.9 ± 5.2 mg of methyl-prednisolone). The mean eosinophil count at baseline was 561 ± 591 cells/µL. The mean eosinophil percentage was 5.3 ± 5.7%. 

The number of asthma exacerbations as well as the mean eosinophil counts decreased, and ACT results improved under daily OCS treatment before initiation of mepolizumab, in comparison to the time before regular OCS intake. There was also a nonsignificant increase in FEV1 values after starting regular daily OCS (Table 3). 

**Table 3:** Comparison of the clinical, laboratory, and functional parameters prior to and after OCS.

N=16	Prior to OCS	Under OCS prior to Mepolizumab	P
Number of asthma exacerbationsin the last 24 weeks, mean ± SD	9.6 ± 8.7	2 ± 2.6	0.001
ACT, mean ± SD	11.9 ± 3.7	18.2 ± 5.5	<0.001
Blood eos %, mean ± SD	13.3 ± 8.9	5.3 ± 5.7	<0.001
Blood eos count mean ± SD	1371 ± 1182	561 ± 591	0.002
FEV1 %, mean ± SD	71.4 ± 19	81 ± 30	0.241
FEV1 L/s, mean ± SD	1920 ± 805	2091 ± 962	0.425

ACT: Asthma control test, eos: eosinophil, OCS: oral corticosteroid

Mepolizumab treatment was found to be very effective in all study subjects. With regard to adverse events, only one patient showed side effects including arthralgia, and malaise occurred on the day after administration of the first two doses. The patient also had a third reaction on the day following administration, and fever, nausea, and vomiting were reported. Mepolizumab was discontinued, and this patient was excluded from the study. The patient was symptom-free during the follow-up period. Further comparisons between study subjects included only the remaining 15 patients. 

When comparing the change in blood eosinophil counts, daily OCS doses, and ACT scores at baseline and week 12 of mepolizumab treatment, a marked decrease in peripheral eosinophil counts (5.5% ± 5.8 vs. 1.3% ± 0.7; P = 0.013) and an increase in ACT scores (18 ± 5.7 vs. 22.5 ± 3.6; P = 0.011) were observed. The OCS dose was decreased in all of the patients; the daily OCS dosage was completely withdrawn in 3 (20%) patients, and 15 out of 15 patients (100%) were classified as treatment responders at week 12. No marked changes in FEV1 values were observed at this time point (80 ± 30.7% vs. 84 ± 26%) (Table 4).

**Table 4:** Comparison of the clinical, laboratory, and functional parameters at the baseline and 12th and 24th weeks.

N=15	Premepolizumab	Mepolizumab12th Week	P*	Mepolizumab24th Week	P**
Methylprednisolone equivalentsystemic steroid dose, mg, mean ± SD	9.2±5.2	2.8±2.2	<0.001	1.3±1.4	<0.001
Number of asthma exacerbationsin the last 24 weeks, mean ± SD	2.1 ± 2.7	0.07 ± 0.26	-	0.07 ± 0.26	0.012
ACT mean ± SD	18 ± 5.7	22.5 ± 3.6	0.011	23.3 ± 3	0.006
Eos %, mean ± SD	5.5 ± 5.8	1.3 ± 0.7	0.013	1.9 ± 1.4	0.029
Eos count mean ± SD	580 ± 607	106 ± 73	0.01	177 ± 137	0.019
FEV1 %, mean ± SD	80 ± 30.7	84 ± 26	0.342	84.6 ± 26	0.392
FEV1 L/s, mean ± SD	2092 ± 995	2156 ± 922	0.434	2232 ± 875	0.533

ACT: Asthma control test, eos: eosinophil. *Comparison of premepolizumab and mepolizumab, 12th week. **Comparison of premepolizumab and mepolizumab, 24th week.

After 24 weeks of mepolizumab treatment, the decrease in blood eosinophil counts (baseline eosinophil count: 5.5 ± 5.8%, 24th week eosinophil count: 1.9 ± 1.4%; P = 0.029) and improvement in ACT scores (baseline ACT: 18 ± 5.7, 24th week ACT: 23.3 ± 3; P = 0.006) continued. The OCS dose was additionally reduced in 9 (60%) patients when compared to 12th week results, and OCS was completely withdrawn in 6 of 15 (40%) patients at week 24. A significant decrease in the 24-week exacerbation rates of pre- and postmepolizumab treatment was observed (2.1 ± 2.7 vs. 0.07 ± 0.26; P = 0.012) (Table 4). Despite the decrease in daily OCS dosages, improvement in all parameters was seen at week 24 under mepolizumab treatment. 

Comparison of OCS dosage, number of asthma exacerbations, ACT, FEV1, and blood eosinophils at the beginning of mepolizumab treatment and at the 12th and 24th weeks is shown in Figure.

During the 6-month mepolizumab treatment, demographics, baseline blood eosinophil counts, exacerbation rates, FEV1 values, and ACT scores were found to be similar regardless of whether OCS was completely withdrawn in the patients. 

On a 10 point NAS, the severity of CRSwNP symptoms decreased from 5.6 ± 4.4 points to 3.2 ± 3.2 points (P = 0.021), and the severity of loss of smell decreased from 4 ± 5.1 points to 2.4 ± 4.2 points (P > 0.05) after 24 weeks of mepolizumab treatment.

## 4. Discussion

This is the first real-life study evaluating the short-term (24 weeks) efficacy of mepolizumab treatment in a specific subphenotype of asthma. Our study showed that in patients with OCS-dependent SEA-CRSwNP phenotype, the OCS dose was decreased in all patients, and 40% of them no longer required OCS after 24 weeks of mepolizumab treatment. In addition to the decrease in daily OCS doses, a decrease in the frequency of exacerbations and an increase in ACT scores were observed. However, there were no significant differences in FEV1 values between week 24 and baseline. 

Both severe eosinophilic asthma and nasal polyposis are characterized by marked local eosinophilic inflammation [19]. IL-5 appears to play a key role in the pathogenesis of CRSwNP and eosinophilic asthma [19–23]. In addition, suggestions were made in the severe asthma guidelines of GINA regarding preferred biologics for the type-2 high asthma phenotype, and it was emphasized that factors determining the response to treatment should be taken into consideration .1 Therapy should be initiated with anti-IL5/anti-IL5R mAbs in patients with severe uncontrolled asthma who have a blood eosinophil count of ≥300/µL. Factors that may predict a good response to anti-IL5/IL5R biologics are defined as follows: (a) higher blood eosinophil count (strongly predictive), (b) more frequent severe exacerbations during the previous year (strongly predictive), (c) adult-onset asthma, (d) nasal polyposis, and e) maintenance with OCS at baseline. The efficacy of mepolizumab has been reported, separately, in OCS-dependent asthma, severe eosinophilic asthma, and CRSwNP [3–5,24–26]. Therefore, the OCS-dependent SEA-CRSwNP phenotype appears to be the most appropriate phenotype for mepolizumab. In our study, all study subjects presented with a history of frequent exacerbations, higher eosinophil levels, OCS-dependency, and CRSwNP. For all of these reasons, we decided to initiate mepolizumab therapy for this subphenotype of asthma.

**Figure:**
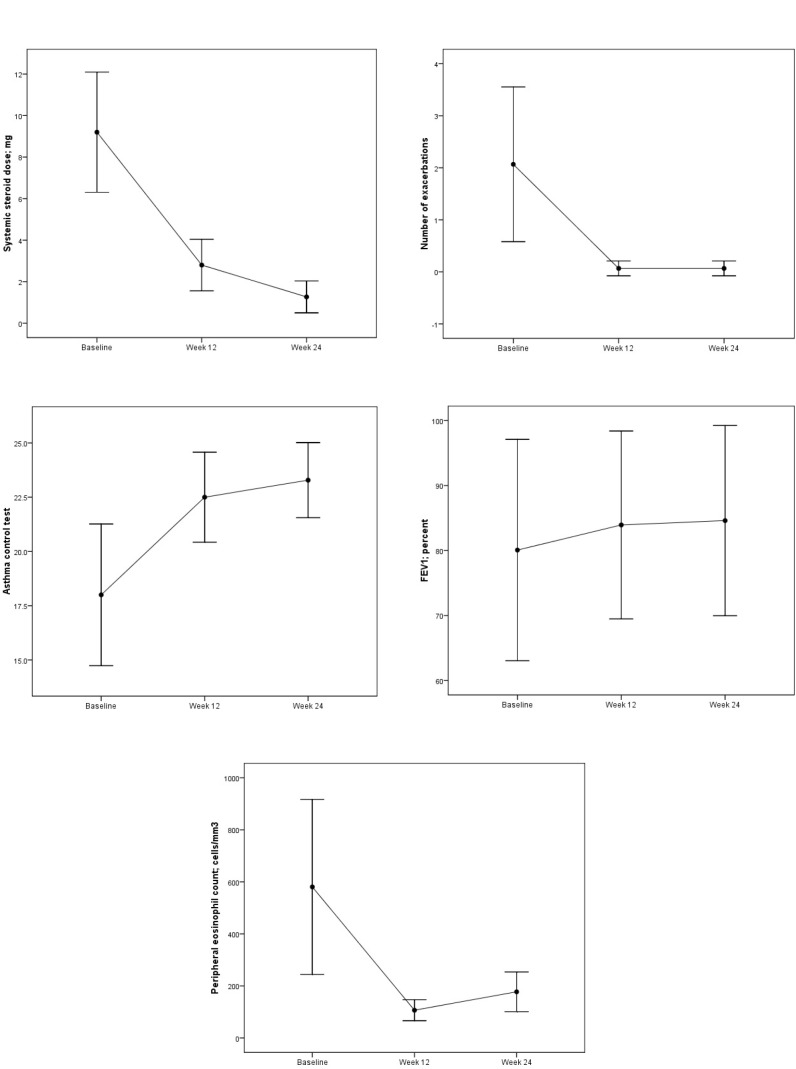
Comparison of OCS dosage (A), number of asthma exacerbations (B), ACT (C), FEV1 (D), and blood eosinophils (E) at the beginning of mepolizumab and at the 12th and 24th weeks after beginning treatment.

One of the main endpoints of all biologics is the withdrawal of the use of OCS in severe asthma [27]. In this real-life study, all patients were prescribed with daily OCS (9.2 ± 5.2 mg of methyl-prednisone, or equivalent). The OCS dose was successfully reduced in all patients, and 40% patients completely discontinued OCS after 24 weeks of mepolizumab therapy. Compared to baseline, at week 24 a significant decrease in the frequency of exacerbations and an increase in ACT scores were observed, in addition to the decrease in daily OCS doses. In the large, placebo-controlled trials, treatment with mepolizumab significantly reduced exacerbation rates and daily OCS doses [3–5]. The Steroid Reduction with Mepolizumab Study (SIRIUS) demonstrated that mepolizumab, in addition to decreasing asthma exacerbations and improving the quality of life, was also capable of significantly diminishing daily OCS intake (reduction: 50%, complete discontinuation: 14%) [3]. However, the OCS reduction (100%) and complete discontinuation (40%) rates in our study were much higher at week 24. This positive outcome could be due to the fact that the add-on therapy with mepolizumab was initiated in a specific eosinophilic asthma subphenotype that may derive a higher potential benefit from mepolizumab therapy. In another real-life study supporting the results of the present study, Pelaia et al. established that OCS could be reduced in all patients with significant reduction in exacerbations after 24 weeks of initiation of mepolizumab therapy in patients with OCS-dependent severe eosinophilic asthma [28]. The common feature of these studies is that all of the patients in both studies were OCS-dependent, and the initial eosinophil counts during OCS therapy were higher (580 ± 607 cells/μL, 647.1 ± 274.7 cells/μL, respectively). However, nasal polyp ratios were not reported in this study. These two real-life studies have shown that the early results from mepolizumab are quite good, especially in patients with high blood eosinophilia and OCS dependency. Prioritizing these patients will be more meaningful in terms of treatment success among patients indicated for mepolizumab therapy.

In the present study, the clinically relevant improvement in asthma control elicited by mepolizumab was paralleled by a prominent and sustained decrease in blood eosinophil counts. This finding confirms the results of several large-cohort and real-life studies [4,28]. Undeniably, blood eosinophils are now considered a reliable biomarker for predicting and assessing the therapeutic efficacy of mepolizumab in severe eosinophilic asthma [29,30]. The initial high eosinophil levels in patients in the present study were associated with a good response to mepolizumab. An increased blood eosinophil count (300 or 400 cells/μL) in asthmatic patients is associated with an increased risk of exacerbations [31,32]. Therefore, one of the goals of mepolizumab treatment that should be emphasized is keeping blood eosinophils below these cut-off values in severe eosinophilic asthma [33]. In the present study, the remarkable decrease in blood eosinophil levels evoked by mepolizumab was paralleled by a reduction in the rate of asthma exacerbation and an improvement in asthma control. 

Contradictory reports on the effects of mepolizumab on FEV1 have been published. Some studies have indicated a modest increase in FEV1 with mepolizumab therapy [5,16,24], while others claim that FEV1 did not improve with the administration of mepolizumab [1,2,4,34]. In the present study, no significant change in FEV1 values was noted after 12 or 24 weeks of mepolizumab treatment, compared to baseline. Yet, the important point here is that there was no deterioration in pretreatment FEV1 values, despite dosage reduction or discontinuation of OCS.

The SIRIUS study, which included OCS-dependent eosinophilic asthma patients, reported the rate of nasal polyps to be 23%. In another study which included OCS-dependent eosinophilic asthma patients, this rate was 30% [3,24]. However, the effect of mepolizumab on nasal polyps was not evaluated in these studies. In a randomized, double-blind, placebo-controlled study recruiting adult patients with recurrent nasal polyposis requiring surgery, patients received 750 mg intravenous mepolizumab. A significant improvement in all individual VAS symptom scores was observed in the mepolizumab group, in comparison to placebo [25]. In the present study, a significant improvement in NAS scores that assessed general CRSwNP symptoms at 24 weeks (rhinorrhea, nasal blockage, postnasal drip, and loss of smell) was observed. However, there was no significant improvement when NAS was evaluated only for smell loss. We speculate that other pathways may be more dominant in the development of eosinophilic inflammation in the nasal polyposis in these subgroups (IL4/IL13 dominant type-2 inflammation) or that the dose of mepolizumab may be insufficient to reduce eosinophilic inflammation at the tissue level (the mepolizumab dose used in the two, abovementioned studies on CRSwNP was 750 mg; the dose used in the present study was 100 mg). 

The major limitations of the present study were the inclusion of a small number of patients and its retrospective design. We could not recruit a high number of patients as the study was conducted in a very specific patient cohort. Although we obtained good clinical results from mepolizumab therapy for a specific asthma subphenotype, a large-scale series is still necessary for robust results. Another limitation was that we only compared asthma exacerbation rates before and after 6 months of mepolizumab initiation. As exacerbations mainly occur during winter, assessment for at least 12 months is necessary to properly reflect the exacerbation rates [35]. However, patients who received mepolizumab for at least 6 months passed through the autumn of 2018 and winter and spring of 2019. In other words, the patients were treated with mepolizumab during the period in which the risk of asthma exacerbation is high, and the results were compared 6 months before mepolizumab initiation. The authors speculate that decreases in exacerbations during a 12-month period may be even greater.

In conclusion, the authors found that subcutaneous mepolizumab administration significantly decreased blood eosinophil levels, asthma exacerbations, and daily OCS doses. These excellent therapeutic effects were associated with a marked improvement in symptoms as well as a very good short-term safety profile for the drug and its tolerability in this single-center study. Therefore, the availability of mepolizumab in daily medical practice undoubtedly represents a valuable advancement in the management of patients with OCS-dependent SEA-CRSwNP. The ideal scenario for therapeutic intervention would be specific selection criteria with a clear prediction of clinical benefits. This small study was clinically directed and highlighted the importance of patient selection for investigating the use of mepolizumab in severe asthma. Nevertheless, further extended real-life investigations are required to confirm the results obtained in our study. 
